# Determinants of access to the SARS-CoV-2 vaccine: a preliminary approach

**DOI:** 10.1186/s12939-021-01520-4

**Published:** 2021-08-14

**Authors:** Brigitte Renata Bezerra de Oliveira, Ana Iza Gomes da Penha Sobral, Marcelo Luiz Monteiro Marinho, Marcos Felipe Falcão Sobral, André de Souza Melo, Gisleia Benini Duarte

**Affiliations:** 1 Departamento de Administração, Federal Rural University of Pernambuco, Avenida Dom Manoel de Medeiros, s/n – Dois Irmãos, Recife, PE Brazil; 2 Departamento de Psicologia Cognitiva, Federal University of Pernambuco, Av. Prof. Moraes Rego, 1235-Cidade Universitária, Recife, PE 50670-901 Brazil; 3 Departamento de Computação, Federal Rural University of Pernambuco, Avenida Dom Manoel de Medeiros, s/n – Dois Irmãos, Recife, PE Brazil; 4 Departamento de Economia, Federal Rural University of Pernambuco, Avenida Dom Manoel de Medeiros, s/n – Dois Irmãos, Recife, PE Brazil

**Keywords:** SARS-CoV-2 Vaccination, COVID-19, Vaccine distribution

## Abstract

**Background:**

The determinants of access to immunizers are still poorly understood, leading to questions about which criteria were considered in this distribution. Given the above, the present study aimed to analyze the determinants of access to the SARS-CoV-2 vaccine by different countries.

**Methods:**

The study covered 189 countries using data from different public databases, and collected until February 19, 2021. We used eight explanatory variables: gross domestic product (GDP), extreme poverty, human development index (HDI), life expectancy, median age, coronavirus disease 2019 (COVID-19) cases, COVID-19 tests, and COVID-19 deaths. The endogenous variables were total vaccine doses, vaccine doses per thousand, and days of vaccination. The structural equation modeling (SEM) technique was applied to establish the causal relationship between the country's COVID-19 impact, socioeconomic variables, and vaccine access. To support SEM, we used confirmatory factor analysis, t-test, and Pearson's correlation.

**Results:**

We collected the sample on February 19, and to date, 80 countries (42.1%) had already received a batch of immunizers against COVID-19. The countries with first access to the vaccine (e.g., number of days elapsed since they took the first dose) were the United Kingdom (68), China (68), Russia (66), and Israel (62). The countries receiving the highest doses were the United States, China, India, and Israel. The countries with extreme poverty had lower access to vaccines and the richer countries gained priority access. Countries most affected by COVID (deaths and cases) also received immunizers earlier and in greater volumes. Unfortunately, similar to other vaccines, indicators, such as income, poverty, and human development, influence vaccines' access. Thus affecting the population of vulnerable and less protected countries. Therefore, global initiatives for the equitable distribution of COVID need to be discussed and encouraged.

**Conclusions:**

Determinants of vaccine distribution consider the impact of the disease in the country and are also affected by favorable socioeconomic indicators. The COVID-19 vaccines need to be accessible to all affected countries, regardless of their social hands.

**Supplementary Information:**

The online version contains supplementary material available at 10.1186/s12939-021-01520-4.

## Introduction

In December 2019, a group of new infectious respiratory syndromes of unknown cause was observed in Wuhan, China [1]. Scientists and doctors worked together and quickly identified the new coronavirus as severe acute respiratory syndrome coronavirus 2 (SARS-CoV-2) [2]. On March 11, 2020, the World Health Organization (WHO) declared COVID-19 a global pandemic [3]. Several countries have relied on prevention strategies to control the pandemic, using non-pharmaceutical interventions to reduce cases including quarantine, social distance, wearing of masks and gloves, washing of hands, etc.

Physical distance measures, which are necessary to prevent the spread of COVID-19, are substantially more difficult for those with adverse social determinants and can contribute to short-and long-term morbidity.

According to the National Commission on Social Determinants of Health (CNDSS) in Brazil, the health social determinants are defined as social, economic, cultural, ethnic/racial, psychological, and behavioral factors that influence health problems and their risk factors in the population [4]. Elderly people and pre-existing diseases are more susceptible to death [5, 6]. Ethnic origin has also been identified as an influencing factor in the infection evolution caused by COVID-19 [7]. The African population tends to have a higher rate of comorbidities, such as hypertension and diabetes. The [8] study addressed the racial relationship and mortality rate for Covid-19 in the United States and showed that the number of deaths of black people is disproportionately higher than that of white people [8].

Another factor that should be highlighted is the availability of resources and access to health systems, differentiating between countries and even between regions according to socioeconomic factors and/or chronic diseases [7, 9].

While individual factors are essential for identifying which individuals within a group are at most significant risk, differences in health levels between groups and countries are more related to other factors, especially equity in income distribution [4].

Homeless people or families are at an increased risk of infection during physical lockdowns, especially if public spaces are closed, resulting in physical crowding that can increase viral transmission and reduced access to care [10]. The effects of social determinants of health and morbidity in COVID-19 may be underestimated. Although COVID-19 has been called a great equalizer, requiring physical distance measures worldwide, it is increasingly evident that social inequalities in health affect the inequality and mortality of COVID-19.

On the other hand, during this period, laboratories developed detection tests and supplied them to the global population [11]. However, since the beginning of the pandemic, most countries still face major challenges in increasing testing capacity, including the lack of understanding of the different types of tests and how they can be used [11].

Much of the pandemic narrative last year was that all hopes of a return to normalcy depended on the development of an effective vaccine [12]. This rhetoric was deaf to the concerns of vaccine and public health experts, and for many a SARS-CoV-2 vaccine has become the hope to get us out of lockdown cycles and economic decline. Against all precedents, by the advent of 2021, the world had in its arsenal several vaccines with proven efficacy against symptomatic COVID-19 [13].

Unfortunately, numerous problems and uncertainties surround COVID-19 vaccines [14]. Supply chain constraints, prices, and unequal vaccine purchases between countries mean that coverage in most, if not all, countries will remain below the level required for herd immunity [15].

WHO has announced that more than 150 countries are engaged in a global consortium that brings together WHO, UNICEF, and other organizations worldwide called Vaccine Global Access (COVAX). This consortium consists of a mechanism designed to guarantee rapid, fair, and equitable access to vaccines worldwide [16]. In general, studies of health inequities assume thar they can be preventable, unfair, and unnecessary [4].

We can say that vaccines will make an important contribution to the return to normalcy. However, they are only part of an exit strategy.

Nonetheless, with the arrival of vaccines, few studies have evaluated the access to these vaccines according to socioeconomic factors [17]. These factors can be particularly important in poor or developing countries, characterized by social inequality, where large populations live below the poverty line and in homes without basic sanitation. The relationship between socioeconomic variables and access to health care is essential. The disadvantaged socioeconomic position (SEP) is widely associated with disease and mortality, and that this is also observed in the COVID 19 pandemic [17]. They emphasize that recommendations and policies need to recognize the collective contribution of socioeconomic determinants and their inter-relationship with clinical variables to mitigate the risk of a pandemic.

For example, data from the University of Oxford's Our World Data database [18] show that 80 out of 189 countries have started their internal vaccination campaigns, which raises the debate on the central reasons for this non-equal distribution and highlights the importance of the country's engagement in enabling access to immunization. Thus, this study aimed to identify the determinants that influence access to the SARS-CoV-2 vaccine by different countries and, consequently, public health during the Covid-19 epidemic.

**Fig. 1:**
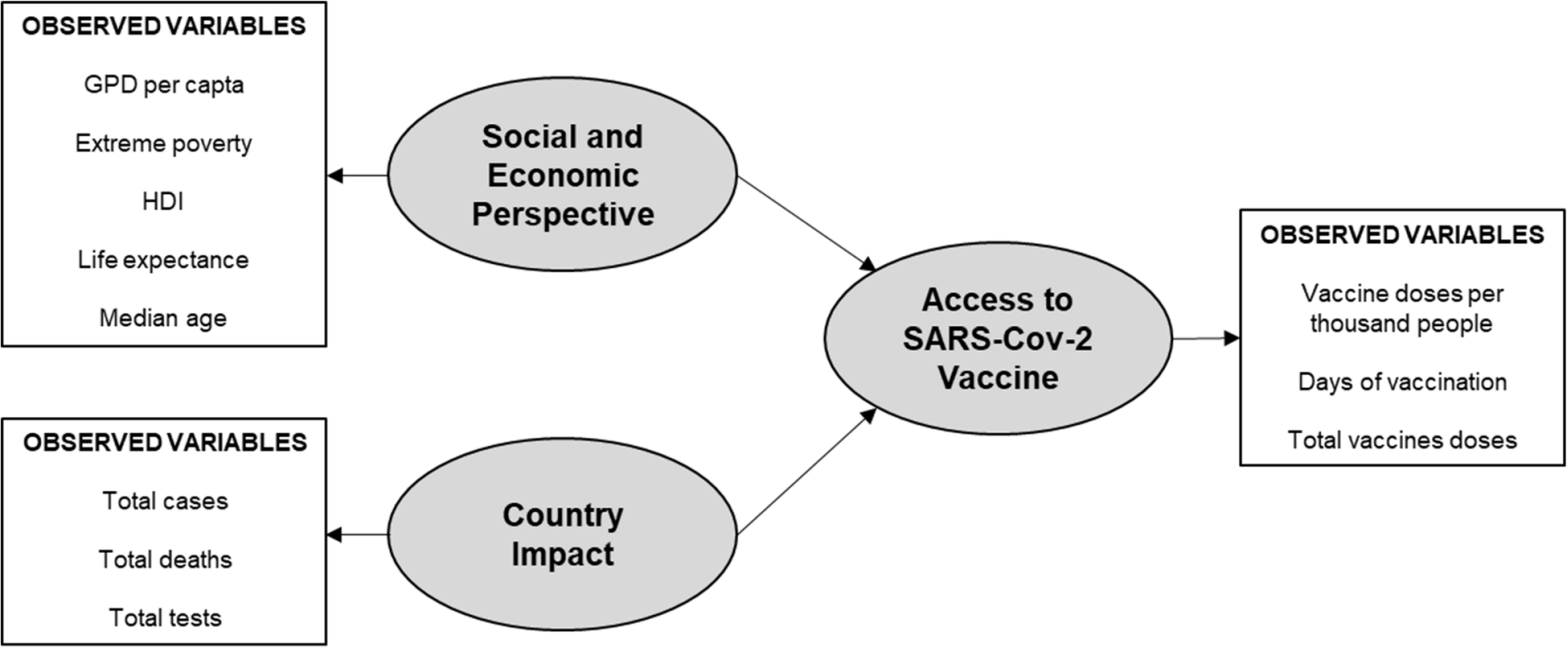
Conceptual proposition

The conceptual proposition is shown in Fig. 1. We assume that GDP per capita and Extreme poverty influence the country's vaccine coverage through the HDI index. The country impact of COVID-19 is represented by the cases, deaths, and tests performed. Our study tests the hypothesis that these variables could influence vaccine access.

## Methods

### Study design and setting

We performed cross-sectional and correlational analyses. The data used were obtained from an aggregated database. Therefore, there was no identification of the subjects or the involvement of human beings or animals. Anonymous data were extracted from databases contained in the database of Our World in Data [18], World Bank Database [19], and World Health Organization [3]. Although countries are identified, patient data is anonymous. We used consolidated data available until February 19, 2021. Data from 189 countries were used in this study. The manuscript structure was presented following the STROBE guidelines for observational studies.

### Data sources, study size, and variables

The research used three data sources, the first being from the Our World in the Vaccines of the University of Oxford Portal [18]. Data were also collected from the World Bank Data Catalog [19] and the World Health Organization [3]. The database was available as diary data in a Microsoft Excel file, which was processed, totaled, and exported to SPSS Statistics 22 and SPSS AMOS.

We used the 11 observed variables in the model: two predictors (gross domestic product [GDP] per capita and extreme poverty), three mediating variables (human development index [HDI], life expectancy, and median age), and three outcome (dependent) variables (days of vaccination, total vaccines doses and vaccine doses per thousand people). To assess the impact of COVID-19, data on the total number of deaths (COVID-19 deaths), total number of cases (COVID-19 cases), and testing (COVID-19 tests) were used to constitute the latent variables or construct "country impact.” All three observed variables represented the cumulative total during the study period.

Access to the vaccine was modeled on three outcome variables: total vaccine doses, vaccine doses per thousand, and days since the first vaccine batch was received (days of vaccination). The response variable was subdivided into three due to differences between countries that received more doses, countries that received vaccines for a longer time, and countries that immunized more quickly.

### Statistical analysis

Data analysis initially determined the psychometric properties and validation of the construct “Country Impact.” We then ran descriptive statistics, normality tests, Pearson's correlation, and factor analysis.

The theoretical model was validated using structural equation modeling (SEM). Structural equations were recognized as an extension of multiple regression. This allowed for the simultaneous estimation of outcome and predictors variables. In addition, it was possible to calculate the corrections between all these variables in a structural structure. It analyzed the causal relationship between the outcome and predictors variables. The interdependent relations are pictorially represented in the so-called path diagram: straight arrows show the impact of VIs (predictors variables) on VDs (outcome variables), and curvilinear arrows demonstrate the covariance between variables.

We implemented structural equation modeling by following the four steps proposed by Hoyle [20]: (i) data collection and preparation: consolidation of the database based on the variables of the theoretical model to be estimated; (ii) specification: definition of the model's operational scheme, identification of parameters, latent variables, regression coefficients, and the correlation between variables; (iii) estimation: a selection of the method based on the nature and distribution of the variable; and (iv) adjustment assessment, checking for the discrepancy between the covariance matrices using the adjustment indices. The model adjustment criteria are listed in Table 1.

**Table 1:** Structural model adjustment criteria

Model adjustment criteria (index)	Definition	Acceptance level	Explication
Chi-square (CMIN) and Chi-square per degrees of freedom (CMIN/*df*)	Tests the H_0_ that the residual covariance estimate is equal to a matrix composed only by zeros. It considers the degrees of freedom (df) of χ^2^. The chi-square value is divided by the number of degrees of freedom to obtain an adjustment value for the model less sensitive to the sample size.	Table value χ^2^; values below 5 are tolerable.	Comparison of values obtained χ^2^ with the tabulated value given by df.
Root square mean error of approximation (RMSEA)	Identify how well a model fits the population, not just the sample used for the estimation.	Ideal values up to 0.08; below 0.10 are tolerable	A value between 0.05 and 0.08 indicates a good approximation
Normed Fit Index (NFI)	Compare the chi-square for the tested model against the chi-square for the baseline model assuming that the measured variables are completely independent.	0 (has no adjustment) and 1 (perfect fit)	A value close to 0.90 or 0.95 reflects a good adjustment
Comparative Fit Index (CFI)	Comparative adjustment index is considered to be more appropriate for the evaluation of models generated from samples considered small	It shows whether and to what extent the quality of adjustment of the proposed model is better than that of the base model.	A value close to 0.90 or 0.95 reflects a good adjustment

## Results

**Fig. 2:**
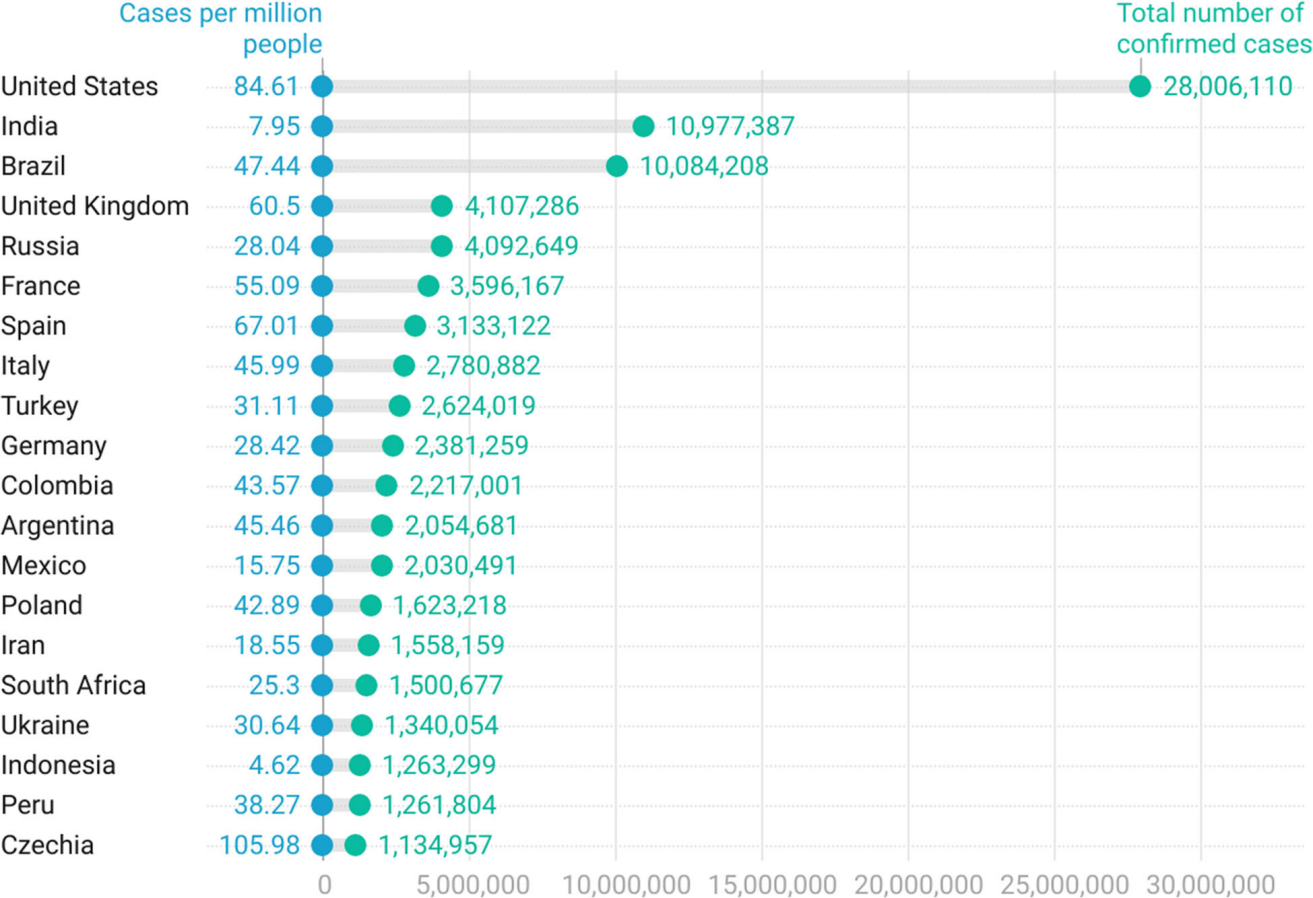
Top 20 countries with COVID-19 cases

**Fig. 3:**
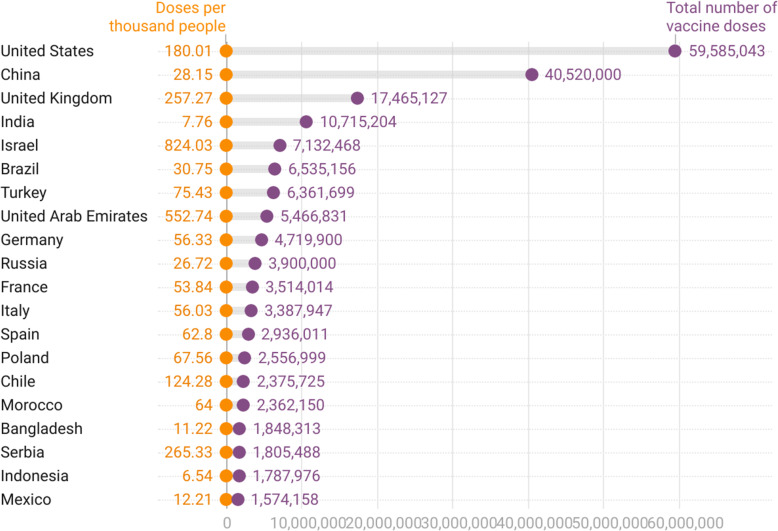
Top 20 countries in the availability of SARS-CoV-2 vaccine doses

The study covered data from 189 countries up to February 19, 2021. The countries with the highest number of confirmed cases (in million) were the United States (28,006), India (10,997), Brazil (10,084), the United Kingdom (4,107), and Russia (4,092). Despite the United States having the highest of absolute COVID-19 cases, they are in the 8th place in terms of cases per million, followed by the United Kingdom (21st), France (25th), Brazil (29th), Russia (62nd), and India (102nd). Czechia ranks 20th in confirmed cases and 3rd in cases per million. Andorra has the highest number of confirmed cases per thousand inhabitants (137.77). The absolute and relative cases are shown in Figs. 2 and 3.

The countries that received the highest doses of vaccine were (in millions): United States (59,585), China (40,520), the United Kingdom (17,465), India (10,715), and Israel (7,132). The top 20 countries in the availability of SARS-CoV-2 vaccine doses and are shown in Fig. 2. In terms of population coverage, Israel had 804.03 doses per 1000 people, followed by Seychelles (623,87), the United Arab Emirates (552,74), Serbia (265,33), and the United Kingdom (257,27). China and Israel were not in the top 20 of confirmed cases in absolute and relative numbers, even though they are among the countries with the highest volume of immunizers available.

By February 19, 2021, 80 countries (42.1%) had already received a batch of immunizers against COVID-19. The first countries to have access to the COVID vaccine were the United Kingdom (68 days), China (68 days), Russia (66 days), Israel (62 days), the United States (61 days), and Bahrain (58 days). The median number of days was 40.5.

We compared the socioeconomic variables among countries that initiated the immunization process and countries that had not yet initiated vaccination, regardless of how long it was (number of days), the total number of doses available, and the population coverage (vaccines per thousand people). The results were significant in a t-test (P≤0,001) to all indicators in the model. Notably, countries that are already vaccinating their population have better socioeconomic indicators, as shown in Table 2 (identified mean for each indicator). About the indicators: (i) GDP per capta is presented in Thousand dollars; (ii) Extreme Poverty is described by the World Bank as extremely poor, living with a daily income of less than $1.90. We can interpret this index as the higher, the worse; (iii) The Human Development Index (HDI) is measured by the United Nations and represents the evolution of countries' social well-being. The index values ​​range from 0 to 1, the closer to 1 the greater human development; (iv) Life Expectancy is presented in Years; and (v) Median age is presented in Years.

**Table 2:** T-test: socioeconomic indicators X immunization initiated

	GDP per capta	Extreme Poverty	HDI	Life Expectancy	Median age
Vaccination initiated	30,167.155	2.704	0.829	77.930	36.534
Vaccination not initiated	9,495.675	22.971	0.641	69.063	25.371

Note: *GDP* gross domestic product, *HDI* human development index

The refinement of the construct Country Impact started with the identification of the medians for each indicator (in thousands): COVID-19 cases (58,546), COVID-19 deaths (1,018), and the country’s total tests (2210,402). The data, as expected due to the nature of the data and the wide diversity of the countries, do not follow a normal distribution, according to the results of asymmetry and kurtosis of the curve, confirmed by the Kolmogorov-Smirnov and Shapiro-Wilk tests, both with *P* <0.001.

The Country Impact construct showed a strong positive correlation with its indicators. Furthermore, given that the analysis was shown to be unifactorial (cumulative variance of 90.29%), it was not possible to present the adjustment indexes of the measurement model (confirmatory factor analysis). Thus, the definition of the latent variable was limited only by internal consistency and subsequent adjustment in the structural model. Furthermore, it was not suggested that the item be removed due to commonalities. The internal consistency of the construct Country Impact presents a positive Pearson correlation between the COVID-19 cases and COVID-19 deaths (r=0.955, P<0.001), COVID-19 cases and total tests (r=0.845, P<0.001), and total tests and COVID-19 deaths (r=0.759, P<0.001).

Next, Pearson's correlation coefficient between socioeconomic indicators (GDP per capita, extreme poverty, HDI, life expectancy, and median age) was calculated. Extreme poverty had a moderate to strong negative correlation with all other variables. The other indicators had positive associations with each other. This analysis allowed for the inclusion of all five variables in the structural model. The syntheses of the correlations are presented in Table 3.

**Table 3:** Pearson Correlation of socioeconomic variables

	Extreme poverty	HDI	Life expectancy	Median age
GDP per capita	-.510^a^	.818^a^	.730^a^	.719^a^
Extreme poverty		-.777^a^	-.752^a^	-.697^a^
HDI			.912^a^	.895^a^
Life expectancy				.829^a^

^a^ Sig 0.01. *GDP* gross domestic product, *HDI* human development index

The variables that cover vaccine access showed positive correlations between them. The total vaccine dose variable presented a positive Pearson correlation between vaccines per thousand (r = 0.243, P <0.001) and days of vaccination (r = 0.352, P <0.001). Vaccines per thousand variables were also positively related to days of vaccination (r = 0.469, P <0.001). The score was slightly below the desirable. However, it was observed when observing them in a structural model as fully endogenous variables.

The initial round of estimation of the structural model of vaccine access did not present an improper solution. Forty parameters were estimated at 77 different moments, which resulted in 37 degrees of freedom and a Chi-square of 187,697. The minimum was reached for the model to be defined (P <0.001). The explained variance of the model, approximated at 44.5%, marginally met the minimum required for the specification. The structural model considered data from all countries in an integrated manner.

The estimation method used was maximum likelihood (ML); for continuous variables and the absence of normality in the data distributions. The generalized least squares (GLS) method was the most suitable [13]. However, it was not possible given the absence of data in some variables and the need to estimate means and intercepts. The ML method was chosen because it is suitable for continuous variables. The values ​​estimated by the ML method aim to produce the values ​​of the equation parameters, minimizing the degree of discrepancy between the observed covariance matrix and that implied in the model, which aids in the best fit. The standardized weights are listed in Table 4.

**Table 4:** Standardized weights of the structural model vaccine Access

Indicator	Path	*β*
Life expectancy	←Extreme Poverty	-.776^a^
HDI	← GDP per capita	.252^a^
Median age	← Life expectancy	-.846^a^
HDI	←Extreme Poverty	-.207^a^
HDI	← Life expectancy	.618^a^
COVID-19 tests	← Country Impact	.922^a^
COVID-19 deaths	← Country Impact	.791^a^
COVID-19 cases	← Country Impact	.838^a^
Total vaccine doses	← Country Impact	.931^a^
Vaccine doses per thousand	← HDI	.280^a^
Days of vaccination	← Median age	.611^a^
Days of vaccination	← Country Impact	.249^a^

^a^ Sig 0.01. *GDP* gross domestic product, *HDI* human development index

The model was considered adjusted with no need for re-specification. The Chi-square per degree of freedom (CMIN/Df) result was 5,073, considered marginally adequate. The results of the normed fit index (NFI) were 0.902 and the comparative fit index (CFI) was 0.921, which demonstrates a good adjustment of the model. The Root Mean Square Error of Approximation (RMSEA) was 0.147, and the ideal result was limited to 0.1. However, this is an indicator that is strongly influenced by the sample size, degrees of freedom of the model, and the estimation method used, especially ML.

**Fig. 4:**
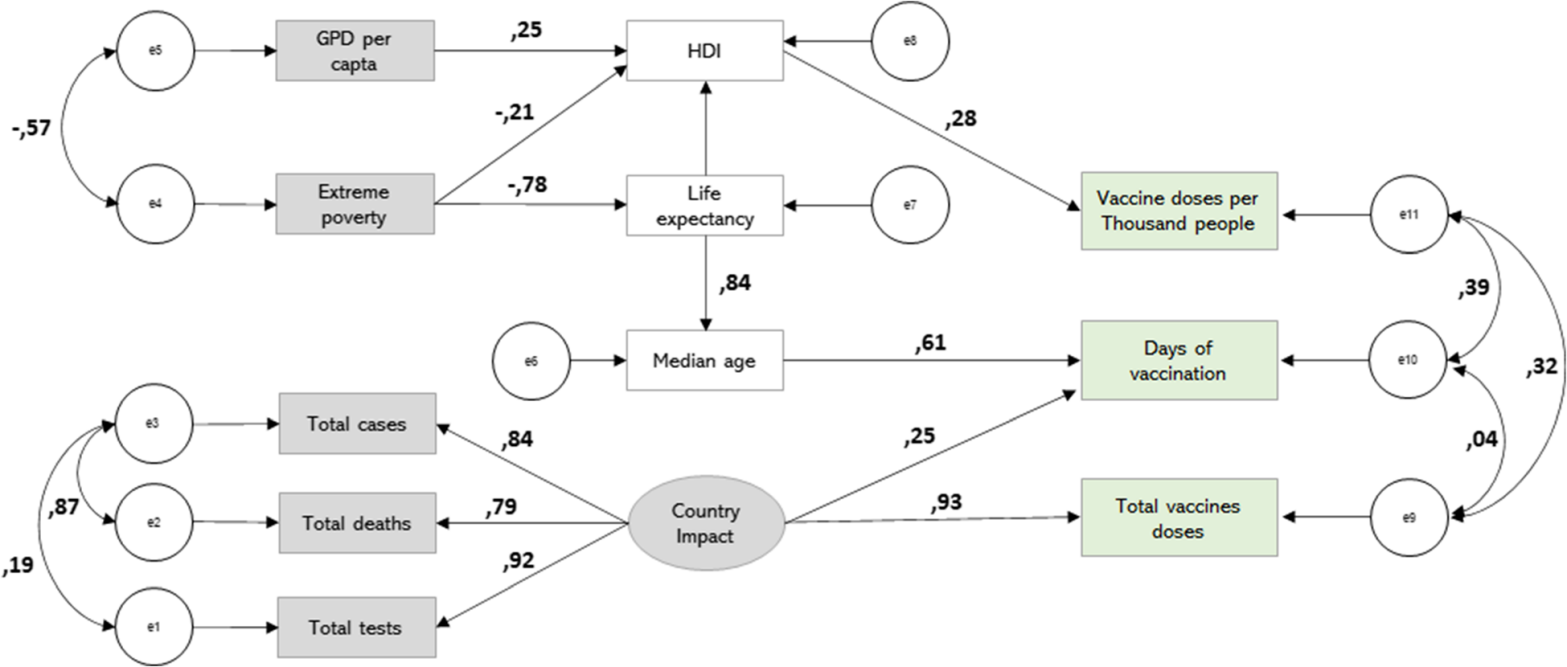
Structural model: Vaccine access

Although we tried to analyze all the countries that had published data, the sample was considered small for SEM and the degrees of freedom of the model were considered low. Therefore, the RMSEA tended to reject models that were acceptable when these restrictions occurred [21, 22]. The structural model is shown in Fig. 4.

The upper section of the model covers the impact of socioeconomic indicators on access to the vaccine. The exogenous background is economic (GDP per capita and extreme poverty). The mediators are the HDI and life expectancy. Life expectancy impacts the vaccine through the median age.

The determinants of the outcome variable vaccine doses per thousand are GDP per capita (positive direction), extreme poverty (negative direction), life expectancy (positive direction), and HDI (direct relationship). For the outcome variable days of vaccination, the determinants are extreme poverty (negative direction), life expectancy, and median age. This relationship had moderate to high weights. In this way, it was possible to affirm that better socioeconomic indicators led to better coverage of the population and priority access to vaccination.

The bottom section of the model assesses Country Impact using absolute numbers. Standardized weights (0.931) and covariance coefficients (0.445) allowed us to state that total vaccine doses are explained by COVID-19 tests, COVID-19 deaths, and COVID-19 cases. The construct also determined, to a lesser extent, the days of vaccination. The low covariance between the two access variables reinforced the argument of analyzing them separately.

We highlight the average variance of 44.5%; this percentage found is justified by the absence of a theoretical basis and previous studies on the determinants of this specific vaccine.

The structural model (Fig. 4) allows the observation of the direct effects (shown in the diagram) and the indirect effects resulting from the multiplication of the direct effects.

In the indirect effects of the model, a negative association was identified between extreme poverty and vaccines (vaccine doses per thousand, and days of vaccination). Thus, a reduction of one standard deviation in extreme poverty was accompanied by an increase of 0.06 deviations in vaccine doses per thousand. The impact was stronger on the days of vaccination indicator, which showed an increase of 0.39. The country's income, measured through GDP per capita, showed a positive association with vaccine doses per thousand, generating an increase of 0.07 standard deviation for each increase in the deviation of per capita income.

In the direct effect, the HDI variable was positively associated with vaccine dose per thousand. For each increase of 1 standard deviation in the HDI, it increased by 0.280 deviations in the vaccine doses per thousand. In absolute numbers, each increment of a unit to the HDI increased the score of the variable vaccine doses per thousand by 171,193. This shows that countries with the highest human development also received a higher volume of vaccine doses/thousand inhabitants.

The vaccine also arrived in higher volumes to countries that experienced the greatest impact from the effects of COVID (deaths, cases, and testing). For each standard deviation in the Country Impact variable, there was an increase in the volume of vaccines received by 0.931. With each increase of one unit in the Country Impact score, there was an increase of 0.167 in the volume of doses received. The country's impact was also positively related to the speed of arrival of the vaccine (0.249 standard deviations). In this way, countries with high numbers of cases and deaths also received the vaccine faster than others.

Finally, the speed of access to the vaccine was also affected by the median age (0.611 standard deviations). Every 1 year added to the median, there was an increase of 1.437 in the number of days that the country had access to the vaccine. In other words, the vaccine distribution was influenced by age, and the population most vulnerable to COVID-19.

## Discussion

In this study, we assessed whether vaccine access depended on the country's impact and socio-economic aspects. For this, we extracted data from 189 countries from the Our World in Data, World Bank Database, and World Health Organization databases. Then, we applied the structural equation modeling (SEM) technique to establish the causal relationship between the background and socioeconomic mediators and the country's impact on access to the vaccine until 02/19/2021. The estimation method used was the maximum likelihood. In support of SEM, factor analysis was also used for the latent variable, t-test, and Pearson's correlation.

Since the identification of the high transmissibility of SARS-CoV-2, efforts by researchers, laboratories, and governments have not been spared in seeking to minimize the contamination and consequences of COVID-19. With this, strategies ranged from health promotion intervention, behavioral guidance with an emphasis on hand and object hygiene, the use of 70% alcohol, and wearing of masks to more restrictive measures, such as quarantine, social isolation, and lockdown.

Previous experiences with SARS-CoV viruses indicate that SARS-CoV-2 shares a 79% identity with the former [23], which greatly facilitated vaccine development in an unprecedented time. Therefore, data on the preclinical development of SARS-CoV vaccine candidates allowed that the initial stage of the SARS-CoV-2 vaccine exploratory project was essentially omitted, considerably shortening the research time [24].

The vaccine is widely considered as one of the strategies that will lead to the possible control of the pandemic effectively [13]. The Food and Drug Administration released a document stating that for the development and licensing of SARS-CoV vaccines-2, an efficiency of at least 50% was necessary [24]. In addition, it emphasized the importance of immunization to cover the global population. This required equitable access to effective vaccines for all countries [13].

Some research indicates that most approved immunizers require two doses, with possible booster doses. Thus, at least 16 billion doses will be needed to meet the global demand [24]. Given that current production capacity is still limited, achieving collective or herd immunity on a global scale of 60%– 80% is a challenge [25].

The financial contribution was the main determinant that allowed the accelerated development of vaccines. However, the process of its distribution to countries is not clear [24]. This is because countries that have allocated resources for research development and/or those that can pay for them in advance may have preferential access [26].

In our study, we identified that countries with better socioeconomic indicators, such as longer life expectancy and higher HDI, obtained priority access to vaccination and better coverage of the population based on the number of doses acquired.

The pandemic had a strong impact on China, the European Union, and the United States, making global political centers put the problem at the center of their concerns [27]. As such, countries such as the United States, Canada, Germany, and some within the European Union have proposed to invest more than a billion dollars in public funds for research, vaccine development, diagnostics, and other promising therapies for COVID-19 [26].

The leadership of these financial contributions was with the United States, which invested US $3.6 billion, allocated to the National Institutes of Health. Exorbitant financial contributions from countries, such as China and the European Union [27], were also observed, causing governments to start financing the construction of manufacturing facilities in companies and buying products that did not exist in their marketable form [27].

Although we cannot assert that there is a direct relationship between the number of investments earmarked for the production of vaccines and access to doses, in this study, we found that 80% of the countries received some dose of immunizer against COVID-19. However, China, Russia, the United States, and Israel were the first countries to have access to the vaccine.

Another important finding of our study is the number of doses of immunizers that countries received. To achieve a more equitable distribution, the number of doses destined for countries should prioritize the rates of the highest number of confirmed cases and/or the highest mortality of the population. In this way, the countries that should receive more doses would be the United States, India, Brazil, and the United Kingdom. Representatively, the Americas (the USA, and Brazil) represent approximately 70.8% of all cases and 61.2% of all deaths reported as of October 2020 [28]. However, from the developed model, we were able to identify the fact that the number of doses destined for a country was determined by socioeconomic factors. Thus, the countries that received the vaccine as a priority were those with better socioeconomic conditions, namely the United States, China, and the United Kingdom.

Following this distribution, India and Israel also received a larger number of doses. It is worth mentioning here that Israel has become a vaccination reference for several countries, based on a global effort and the strategy it has developed to immunize the entire population [29].

This information corroborates our findings from the moment when we identified a greater population coverage in Israel with approximately 804 doses of vaccines for every thousand inhabitants. Some factors are attributed to the success of Israel's vaccination plan, such as the small number of inhabitants (9.3 million), which led to a small acquisition of vaccine doses; the fact that its population is relatively young, facilitating the policy of prioritizing the immunization of the elderly; and mainly, the mass vaccination plan [30].

Concerns about access to the vaccine by socioeconomically less favored countries have already been raised in a study [31]. This was because of the experience arising from the H1N1 virus pandemic a decade ago, in which countries that did not have the resources to acquire the immunization doses were disadvantaged, being the last to have access [28].

Thus, the inability of low- and middle-income countries, which have limited budgets, introduces an ethical dilemma of access to an essential input for countries that cannot afford to buy it [26]. In our model, we identified extreme poverty as one of the determining factors for access to the vaccine and the immunization of the population.

As a result, the WHO entered into a partnership with a philanthropic organization called GAVI - Global Alliance for Vaccine and Immunization. This was aimed to expand access to vaccines for the poor and very poor countries. From this partnership, Covax (Global Vaccine Access Facility - Covax Facility) was developed. It proposes the rapid development of vaccines against SARS-CoV-2 and its availability to all countries, enabling subsidies and vaccine donations for poor and very poor countries. It also seeks to make vaccine prices affordable [27].

As of July 15, 2020, 75 middle-and, upper-income countries and 90 poor or very poor countries had joined the agreement, which together represents more than 60% of the world population, with representatives from all continents and more than half of the economies [28]. Brazil, which is one of the countries in Latin America with the highest number of infected people and a high mortality rate, is one of the 75 countries adhering to the Covax Facility, establishing an agreement for the purchase of 30.4 million doses of vaccines [27].

In our model, we identified that the HDI correlated positively with the number of doses available per person, corroborating with the study that presented data from 19 countries on personal acceptance of the vaccine. The results indicate that the desire to have a COVID-19 vaccine is far from universal and that this acceptance is influenced by factors, such as education, age, income, and demographic region. Older adults, with higher education, higher income, and of Asian origin, have a greater acceptance of the vaccine [32].

We emphasize that the vaccination programs do not aim to reach 100% of the population due to several factors [25]. However, the vaccination hesitation constitutes an important barrier for the absorption of the vaccine and for the achievement of collective immunity, which is necessary to protect the most vulnerable populations. Therefore, immunity in the herd is reached from a vaccination percentage of the total population.

In this way, the results of the present study represent an alert for country leaders and government officials, as well as public policymakers, to focus attention on socioeconomically disadvantaged populations, to ensure high vaccine and equitable coverage that prevents the spread of SARS-CoV -2 and puts an end to the pandemic.

## Conclusions

The COVID-19 pandemic has put tremendous pressure on the development of safe and effective vaccines involving efforts by diverse researchers, countries, and governments. Until then, information about the aspects of the distribution of these vaccines to countries and the number of doses that each one should receive was not clear and is still scarce in the literature.

Therefore, based on a structural model, with the total number of doses received as determinants, this study identified that the distribution was influenced by the socioeconomic conditions of the countries. Countries with a higher number of transmissions, higher mortality rate, more vulnerable population group (older age), and better socioeconomic indicators obtained priority access to vaccination, a higher number of doses, and consequently, better vaccination coverage.

Although there are some limitations, such as the number of doses required to immunize the entire population, the scientific findings in this study contribute information that can support decision-making regarding more equitable distribution of vaccines to countries and the number of doses required to immunize the entire population. Thus, these results showed that public health policies must be attentive to the marks of social and economic inequalities in the countries to coordinate efforts between different governmental spheres so that the end of the pandemic happens as quickly as possible.

Future research must be conducted in order to understand some other determinants of access to the vaccine. This includes political and legal aspects, production and distribution infrastructure of the country, types of immunizers and their manufacturers, as well as behavioral and cultural issues and the acceptance of the immunization process by the population.

## Supplementary Information

**Additional file 1.** Countries considered in sample and days of SARS-COv-2 vaccination until February 19th.

**Additional file 2.** Frequency distribution of the variables analyzed in the study.

## Data Availability

The datasets generated during and/or analysed during the current study are available in the Our World in Data repository [https://ourworldindata.org/covid-vaccinations].

## Abbreviations

Covax: Global vaccine access
COVID-19: Coronavirus disease 2019
GAVI: Global alliance for vaccine and immunization
GDP: Gross domestic product
HDI: Human development index
SARS-CoV-2: Severe acute respiratory syndrome coronavirus 2
SEM: Structural equation modelling
STROBE: Strengthening the Reporting of Observational studies in Epidemiology
VDs: Outcome variables
Vis: Predictors variables
WHO: World Health Organization
