# Supplementary material for: Determinants of access to the SARS-CoV-2 vaccine: a preliminary approach
Source: Int J Equity Health. 2021 Aug 14;20:183. doi: 10.1186/s12939-021-01520-4 (PMC8363862; doi:10.1186/s12939-021-01520-4)
Supplement: Supplementary file 1 — Additional file 1. Countries considered in sample and days of SARS-COv-2 vaccination until February 19th. [file 12939_2021_1520_MOESM1_ESM.docx]

Appendix 1

Countries considered in sample and days of SARS-COv-2 vaccination until February 19th

|  | **Country** | Days |  | Country | Days |
| --- | --- | --- | --- | --- | --- |
| 1 | Afghanistan | 0 | 48 | Djibouti | 0 |
| 2 | Albania | 38 | 49 | Dominica | 0 |
| 3 | Algeria | 20 | 50 | Dominican Republic | 2 |
| 4 | Andorra | 25 | 51 | Ecuador | 28 |
| 5 | Angola | 0 | 52 | Egypt | 20 |
| 6 | Antigua and Barbuda | 0 | 53 | El Salvador | 0 |
| 7 | Argentina | 52 | 54 | Equatorial Guinea | 0 |
| 8 | Armenia | 0 | 55 | Eritrea | 0 |
| 9 | Australia | 0 | 56 | Estonia | 53 |
| 10 | Austria | 37 | 57 | Eswatini | 0 |
| 11 | Azerbaijan | 13 | 58 | Ethiopia | 0 |
| 12 | Bahamas | 0 | 59 | Fiji | 0 |
| 13 | Bahrain | 58 | 60 | Finland | 50 |
| 14 | Bangladesh | 23 | 61 | France | 54 |
| 15 | Barbados | 4 | 62 | Gabon | 0 |
| 16 | Belarus | 0 | 63 | Gambia | 0 |
| 17 | Belgium | 53 | 64 | Georgia | 0 |
| 18 | Belize | 0 | 65 | Germany | 54 |
| 19 | Benin | 0 | 66 | Ghana | 0 |
| 20 | Bhutan | 0 | 67 | Greece | 53 |
| 21 | Bolivia | 21 | 68 | Grenada | 0 |
| 22 | Bosnia and Herzegovina | 0 | 69 | Guatemala | 0 |
| 23 | Botswana | 0 | 70 | Guinea | 0 |
| 24 | Brazil | 33 | 71 | Guinea-Bissau | 0 |
| 25 | Brunei | 0 | 72 | Guyana | 4 |
| 26 | Bulgaria | 52 | 73 | Haiti | 0 |
| 27 | Burkina Faso | 0 | 74 | Honduras | 0 |
| 28 | Burundi | 0 | 75 | Hungary | 53 |
| 29 | Cambodia | 9 | 76 | Iceland | 51 |
| 30 | Cameroon | 0 | 77 | India | 34 |
| 31 | Canada | 38 | 78 | Indonesia | 28 |
| 32 | Cape Verde | 0 | 79 | Iran | 10 |
| 33 | Central African Republic | 0 | 80 | Iraq | 0 |
| 34 | Chad | 0 | 81 | Ireland | 50 |
| 35 | Chile | 57 | 82 | Israel | 62 |
| 36 | China | 66 | 83 | Italy | 54 |
| 37 | Colombia | 2 | 84 | Jamaica | 0 |
| 38 | Comoros | 0 | 85 | Japan | 2 |
| 39 | Congo | 0 | 86 | Jordan | 0 |
| 40 | Costa Rica | 57 | 87 | Kazakhstan | 0 |
| 41 | Cote d'Ivoire | 0 | 88 | Kenya | 0 |
| 42 | Croatia | 51 | 89 | Kuwait | 53 |
| 43 | Cuba | 0 | 90 | Kyrgyzstan | 0 |
| 44 | Cyprus | 44 | 91 | Laos | 0 |
| 45 | Czechia | 54 | 92 | Latvia | 32 |
| 46 | Democratic Republic of Congo | 0 | 93 | Lebanon | 0 |
| 47 | Denmark | 54 | 94 | Lesotho | 0 |

|  | Country | Days |  | Country | Days |
| --- | --- | --- | --- | --- | --- |
| 95 | Liberia | 0 | 142 | Saint Lucia | 0 |
| 96 | Libya | 0 | 143 | Saint Vincent and the Grenadines | 0 |
| 97 | Liechtenstein | 16 | 144 | Samoa | 0 |
| 98 | Lithuania | 54 | 145 | San Marino | 0 |
| 99 | Luxembourg | 51 | 146 | Sao Tome and Principe | 0 |
| 100 | Madagascar | 0 | 147 | Saudi Arabia | 44 |
| 101 | Malawi | 0 | 148 | Senegal | 0 |
| 102 | Malaysia | 0 | 149 | Serbia | 42 |
| 103 | Maldives | 17 | 150 | Seychelles | 37 |
| 104 | Mali | 0 | 151 | Sierra Leone | 0 |
| 105 | Malta | 33 | 152 | Singapore | 39 |
| 106 | Marshall Islands | 0 | 153 | Slovakia | 45 |
| 107 | Mauritania | 0 | 154 | Slovenia | 54 |
| 107 | Mauritius | 0 | 155 | Solomon Islands | 0 |
| 109 | Mexico | 57 | 156 | Somalia | 0 |
| 110 | Micronesia | 0 | 157 | South Africa | 1 |
| 111 | Moldova | 0 | 158 | South Korea | 0 |
| 112 | Monaco | 32 | 159 | South Sudan | 0 |
| 113 | Mongolia | 0 | 160 | Spain | 46 |
| 114 | Montenegro | 0 | 161 | Sri Lanka | 21 |
| 115 | Morocco | 20 | 162 | Sudan | 0 |
| 116 | Mozambique | 0 | 163 | Suriname | 0 |
| 117 | Myanmar | 23 | 164 | Sweden | 54 |
| 118 | Namibia | 0 | 165 | Switzerland | 26 |
| 119 | Nepal | 18 | 166 | Syria | 0 |
| 120 | Netherlands | 26 | 167 | Taiwan | 0 |
| 121 | New Zealand | 0 | 168 | Tajikistan | 0 |
| 122 | Nicaragua | 0 | 169 | Tanzania | 0 |
| 123 | Niger | 0 | 170 | Thailand | 0 |
| 124 | Nigeria | 0 | 171 | Timor | 0 |
| 125 | North Macedonia | 0 | 172 | Togo | 0 |
| 126 | Norway | 54 | 173 | Trinidad and Tobago | 0 |
| 127 | Oman | 53 | 174 | Tunisia | 0 |
| 128 | Pakistan | 9 | 175 | Turkey | 36 |
| 129 | Palestine | 0 | 176 | Uganda | 0 |
| 130 | Panama | 30 | 177 | Ukraine | 0 |
| 131 | Papua New Guinea | 0 | 178 | United Arab Emirates | 45 |
| 132 | Paraguay | 0 | 179 | United Kingdom | 68 |
| 133 | Peru | 9 | 180 | United States | 61 |
| 134 | Philippines | 0 | 181 | Uruguay | 0 |
| 135 | Poland | 53 | 182 | Uzbekistan | 0 |
| 136 | Portugal | 53 | 183 | Vanuatu | 0 |
| 137 | Qatar | 22 | 184 | Vatican | 0 |
| 138 | Romania | 53 | 185 | Venezuela | 0 |
| 139 | Russia | 66 | 186 | Vietnam | 0 |
| 140 | Rwanda | 0 | 187 | Yemen | 0 |
| 141 | Saint Kitts and Nevis | 0 | 188 | Zambia | 0 |
|  |  |  | 189 | Zimbabwe | 0 |
