# Supplementary material for: Determinants of access to the SARS-CoV-2 vaccine: a preliminary approach
Source: Int J Equity Health. 2021 Aug 14;20:183. doi: 10.1186/s12939-021-01520-4 (PMC8363862; doi:10.1186/s12939-021-01520-4)
Supplement: Supplementary file 2 — Additional file 2. Frequency distribution of the variables analyzed in the study. [file 12939_2021_1520_MOESM2_ESM.docx]

Appendix 2

Frequency distribution of the variables analyzed in the study

| **Statistics** | | | | | | | | | | | | |
| --- | --- | --- | --- | --- | --- | --- | --- | --- | --- | --- | --- | --- |
|  | | Days of vaccination | Vaccine doses per thousand | Total vaccine doses | Total Confirmed Cases | Total Deaths | Total Tests | GPD per capta | Extreme poverty | HDI | Life Expectancy | Median age |
| N | Valid | 189 | 189 | 189 | 189 | 178 | 109 | 181 | 123 | 184 | 189 | 181 |
|  | Missing | 0 | 0 | 0 | 0 | 11 | 80 | 8 | 66 | 5 | 0 | 8 |
